# Investigation of CIP quality in over-the-counter drug stores of resource-limited countries: a comparative study in Vietnam and Nigeria

**DOI:** 10.1093/jacamr/dlaf042

**Published:** 2025-03-19

**Authors:** Tran Nguyen Minh Thu, Itunuoluwa Celestina Oyelayo, Alexa Purgreth, Thi Thanh Xuan Ngo, Adekunle Olugbenga Olowe, Alena Soboleva, Marvin Hempel, Ludger A Wessjohann, Thi Bao Chi Le, Olusola Ojurongbe, Thirumalaisamy P Velavan, Dennis Nurjadi

**Affiliations:** Institute of Medical Microbiology, University of Lübeck and University Hospital Schleswig-Holstein Campus Lübeck, Ratzeburger Allee 160, Lübeck 23562, Germany; Department of Microbiology, University of Medicine and Pharmacy, Hue University, Hue, Vietnam; Department of Medical Microbiology & Parasitology, Ladoke Akintola University of Technology, Ogbomoso, Nigeria; Humboldt Research Hub-Center for Emerging & Re-emerging Infectious Diseases (HRH-CERID), Ladoke Akintola University of Technology, Ogbomoso, Nigeria; Vietnamese-German Center for Medical Research (VG-CARE), Hanoi, Vietnam; Institute of Tropical Medicine, University of Tübingen, Tübingen, Germany; Center of Drug, Cosmetic and Food Quality Control, Hue, Vietnam; Department of Medical Microbiology & Parasitology, Ladoke Akintola University of Technology, Ogbomoso, Nigeria; Humboldt Research Hub-Center for Emerging & Re-emerging Infectious Diseases (HRH-CERID), Ladoke Akintola University of Technology, Ogbomoso, Nigeria; Department of Bioorganic Chemistry, Leibniz Institute of Plant Biochemistry (IPB), Halle (Saale), Germany; Department of Bioorganic Chemistry, Leibniz Institute of Plant Biochemistry (IPB), Halle (Saale), Germany; Department of Bioorganic Chemistry, Leibniz Institute of Plant Biochemistry (IPB), Halle (Saale), Germany; Department of Microbiology, University of Medicine and Pharmacy, Hue University, Hue, Vietnam; Department of Medical Microbiology & Parasitology, Ladoke Akintola University of Technology, Ogbomoso, Nigeria; Humboldt Research Hub-Center for Emerging & Re-emerging Infectious Diseases (HRH-CERID), Ladoke Akintola University of Technology, Ogbomoso, Nigeria; Vietnamese-German Center for Medical Research (VG-CARE), Hanoi, Vietnam; Institute of Tropical Medicine, University of Tübingen, Tübingen, Germany; Faculty of Medicine, Duy Tan University, Da Nang, Vietnam; German Center for Infection Research (DZIF), Partner Site Tübingen, Tübingen, Germany; Institute of Medical Microbiology, University of Lübeck and University Hospital Schleswig-Holstein Campus Lübeck, Ratzeburger Allee 160, Lübeck 23562, Germany; German Center for Infection Research (DZIF), Partner Site Hamburg-Lübeck-Borstel-Riems, Hamburg-Lübeck-Borstel-Riems, Lübeck, Germany

## Abstract

**Introduction:**

CIP, a broad-spectrum antibiotic, is crucial for managing bacterial infections. Its efficacy relies on maintaining high-quality standards, which can be affected by manufacturing, regulatory oversight and storage practices. This study compares the quality of CIP preparations in Vietnam and Nigeria, two nations with contrasting regulatory frameworks, to assess compliance with pharmaceutical standards and identify risks from substandard or falsified medicines.

**Methods:**

A total of 46 CIP preparations were analysed, 20 purchased from 13 vendors in Vietnam and 26 from 13 vendors in Nigeria. Data on vendor qualifications and storage conditions were collected. Antibacterial activity was tested using a modified disk diffusion assay, and content and purity were evaluated via reversed-phase HPLC.

**Results:**

Vietnam's drug outlets showed stricter regulation, with 100% registration and 61.5% staffed by Bachelor of Pharmacy holders, compared with only 23.1% in Nigeria. Temperature and humidity monitoring was universal in Vietnam but minimal in Nigeria (23.1% and 15.4%, respectively). Antimicrobial testing confirmed effectiveness for all but one sample (Vietnam), while Nigerian samples had greater variability. Reversed-phase HPLC revealed seven Nigerian samples (26.9%) with <80% declared CIP content, all from one manufacturer. Median content was 91% in Vietnam and 88% in Nigeria, with most samples meeting purity standards.

**Conclusions:**

The study highlights significant disparities in the regulation, storage practices and quality of CIP between Vietnam and Nigeria. These findings underscore the critical need for improved regulation, monitoring and enforcement in countries with weaker pharmaceutical oversight to ensure drug efficacy and safety.

## Introduction

Antimicrobial resistance (AMR) is an ongoing major global public health threat, with estimated deaths of nearly 5 million annually from bacterial infections alone.^1^ This issue disproportionately impacts low- and middle-income countries (LMICs), particularly in sub-Saharan Africa.^1^ While the focus often centres on factors like inappropriate antibiotic use and poor patient adherence, the problem of low-quality antimicrobials is frequently overlooked.^2^

CIP, categorized within the Watch group of antibiotics, is critical for stewardship programs and ongoing monitoring.^3^ As a broad-spectrum fluoroquinolone, it effectively targets Gram-negative and Gram-positive pathogens, treating infections ranging from urinary tract to skin and bone infections. Despite being prescription-only, enforcement lapses have led to its widespread availability without prescriptions in many countries.

In LMICs, community drug outlets often serve as the first point of healthcare access, yet they are poorly regulated, with inadequately trained sales staff. Previous studies show that over 80% of antibiotics were dispensed without prescriptions in Vietnam and Nigeria.^4,5^ Given the easy access to oral antibiotics,^6,7^ our study aims to evaluate and compare the quality of over-the-counter (OTC) CIP in Vietnam and Nigeria, and determine whether substandard preparations are dispensed without prescription in both countries. The data generated from this study may be useful in informing strategies to combat AMR in Vietnam and Nigeria.

## Methods

### Study design and sample

**Figure 1.**
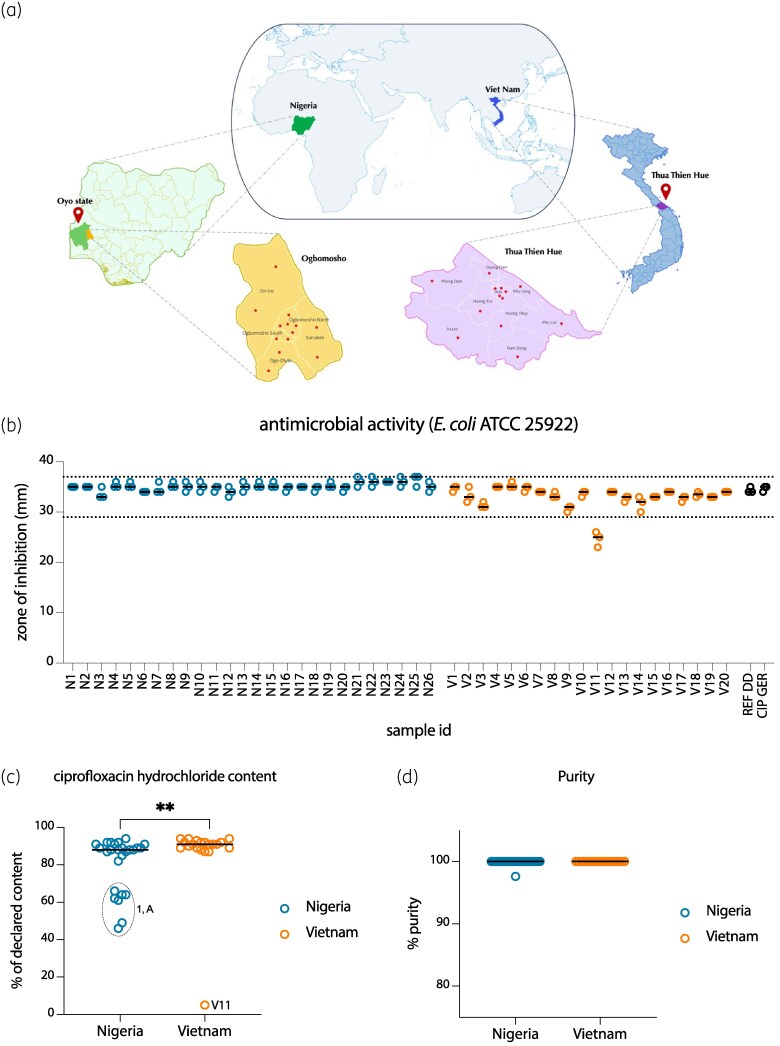
Overview of sampling sites. (a) The dots indicate the location of the vendors included in this study. At least one vendor per district was chosen. (b) The antimicrobial activity of the various CIP preparations was evaluated through disk diffusion using blank disks impregnated with 5 µg of CIP. The dashed horizontal line indicates the acceptable quality control range established by EUCAST for *E. coli* ATCC^®^25922. Reference disk diffusion was performed using a commercially obtained CIP disk (5 µg). CIP hydrochloride content (c) and purity (d) were assessed using HPLC, expressed as a percentage of CIP hydrochloride content in the packaging insert specifications. The substance with the highest deviation from the declared content originated from one manufacturer (1, A). Sample V11 was not soluble in the solvent. *P* values were calculated using the rank-sum test for non-parametric data, with **P < 0.01.

This cross-sectional comparative study assessed the quality of CIP procured from both authorized and unauthorized pharmacies in Vietnam and Nigeria. In Vietnam, the research was conducted in Thua Thien Hue province. CIP preparations were randomly obtained from 13 vendors across the province, including five in Hue City and one from each other towns and districts. In Nigeria, the study took place in Ogbomoso, Oyo State. Twenty-six CIP samples were randomly collected from five vendors in Ogbomoso North and two vendors in each of the other four local government areas in October 2023 (Figure 1a). The study encompassed a range of drug outlets selling oral CIP, with selections made based on the availability of various pharmaceutical brands, dosage forms and strengths. All available CIP samples from each selected pharmacy were collected for subsequent analysis.

### Data collection

Data collection involved a detailed questionnaire that captured information about the pharmacies and the drug storage conditions. Before the data collection and sample procurement, all outlet owners were fully informed about the study’s objectives and the data collection process and signed a consent form. Participation was voluntary, and participants could withdraw from the study at any time without facing any consequences.

### Modified disk diffusion test

Antibiotic solutions were prepared using 0.1 M HCl as the solvent. Tablets from each brand were dissolved in 50 mL of sterile 0.1 M HCl to create 10 mg/mL stock solutions. For quality control, 10 mg standard CIP powder (Sigma-Aldrich, Germany) was similarly dissolved. Blank disks (BD Diagnostics, Germany) were impregnated with 5 µg CIP and dried under sterile conditions before being placed onto Mueller–Hinton agar streaked with 0.5 McFarland *Escherichia coli* ATCC^®^25922 in 0.9% NaCl. As a reference disk diffusion test, a commercially available CIP disk was used (Mast Diagnostics, Germany). The zone of inhibition was measured after an overnight incubation at 37°C.

### Determination of CIP content and purity by HPLC

Three replicates of each CIP tablet (500 mg) were analysed. Analytical reversed-phase HPLC (RP-HPLC) was conducted using an Agilent 1260/1290 system equipped with a quaternary pump, autosampler, thermostated column compartment, fluorescence detector and diode array detector (λ = 278 nm). A LiChroCART C18ec column (250 mm × 4 mm, 5 μm particle size) was utilised. The mobile phases included H_3_PO_4_ (0.025 M) + TEA (pH = 3.0 ± 0.1) and acetonitrile, operated under an isocratic system (0–30 min, 13% B) with a flow rate of 1.5 mL/min, all controlled by Agilent Chemstation software. A detailed description of the methods, including the dissolution process, is included in the Supplementary Methods (available as Supplementary data at *JAC-AMR* Online).

### Data analysis

Data visualization was performed using GraphPad Prism 9 version 9.5.1. The study area map was created using QGIS version 3.34.3. SPSS version 26 was used for the statistical evaluations and descriptive statistical analyses. Statistical significance was determined at *P* < 0.05.

### Ethics

Not required since this work does not involve samples from human or animals.

## Results

### Baseline characteristics of drug outlets

The regulation of drug outlets in Vietnam is notably more stringent than in Nigeria (Table 1). In Vietnam, 62% (*n* = 8) of these outlets function as licensed drug stores, whereas in Nigeria, a significant portion (39%) falls under the ‘other’ category, which includes kiosks and wholesale operations. All drug outlets in Vietnam are registered, while approximately half (*n* = 6/13) in Nigeria remain unregistered. In terms of personnel qualifications, 62% of staff in Vietnam possess a Bachelor of Pharmacy, compared with just 23% in Nigeria, where 31% have an intermediate diploma or an unknown qualification. Storage practices also reflect these differences: 100% of Vietnamese outlets actively monitor temperature and humidity, in contrast to only 23% and 15%, respectively, in Nigeria (*P* < 0.001). Additionally, nearly all outlets in both countries take precautions to protect antibiotics from sunlight (Table 1).

**Table 1.** Characteristics of drug outlets in Nigeria and Vietnam

	Country		*P* value
	Vietnam (*n* = 13)	Nigeria (*n* = 13)	
Type of business			0.040^a^
Pharmacy	5 (38.5%)	4 (30.8%)	
Drug store	8 (61.5%)	4 (30.8%)	
Others	0 (0.0%)	5 (38.5%)	
License for pharmacy business			0.015^b^
Unregistered drug outlets	0 (0%)	6 (46.2%)	
Registered drug outlets	13 (100%)	7 (53.8%)	
Qualification of the person in charge			0.009^a^
Bachelor of pharmacy	8 (61.5%)	3 (23.1%)	
College degree	5 (38.5%)	2 (15.4%)	
Intermediate diploma	0 (0%)	4 (30.8%)	
Unknown qualification	0 (0%)	4 (30.8%)	
Storage condition^c^			
Temperature monitoring			
Yes	13 (100%)	3 (23.1%)	<0.001^b^
No	0 (0%)	10 (76.9%)	
Humidity monitoring			
Yes	13 (100%)	2 (15.4%)	<0.001^b^
No	0 (0%)	11 (84.6%)	
Shielded from sunlight			
Yes	13 (100%)	12 (92.3%)	1.000^b^
No	0 (0%)	1 (7.7%)	

^a^ *P* values by χ² test.

^b^ *P* values by Fisher’s exact test.

^c^Air conditioning was included in the questionnaire, but was not included in the analysis due to many missing data.

### Determination of antimicrobial activity

We evaluated the antimicrobial activity of CIP using a modified Kirby–Bauer disk diffusion test with blank disks impregnated with 5 µg of CIP. Reference standards included a commercially available disk and CIP hydrochloride powder from Germany. Forty-six preparations were tested, including 26 from Nigeria and 20 from Vietnam. All CIP preparations, except the insoluble V11, demonstrated antibacterial activity, with zone diameters within the EUCAST-recommended range of 29–37 mm for *E. coli* ATCC^®^25922 using a 5 µg CIP disk (Figure 1b).

### CIP solubility, content and purity

Following European Pharmacopoeia standards, RP-HPLC analysis at 278 nm was conducted to assess the CIP content and purity. This analysis revealed two significant irregularities. Sample V11 (QLT037) from Vietnam could not be extracted due to unusual additives, rendering it non-compliant with European Pharmacopoeia standards (Figure S1). Additionally, preparation N4 (QLT004) from Nigeria contained tablets of varying colours (brownish) from the same blister packaging, which was atypical (Figure S2).

All samples from Vietnam had at least 87% of the declared content, except the insoluble V11. In Vietnam, the median deviation was 9% (range: 6%–13%), excluding the insoluble sample V11 (Figure 1c). Among the 26 samples from Nigeria, 7 (26.9%) contained <80% of the declared CIP hydrochloride content. The median deviation in CIP hydrochloride content compared with the packaging insert for samples obtained in Nigeria was 12% (range: 6%–54%). Notably, all seven preparations originated from the same manufacturer (Brand 1, Manufacturer A, Figure 1 and Table S1) with different lot numbers. Nearly all samples exhibited a purity of 100%, except for N4, which had a purity of 98%, likely due to the atypical brownish colour of the tablets. Interestingly, this brown discolouration was also observed in one of Brand 1 preparations, which showed the highest deviation from the declared content (Figure 1d).

## Discussion

Our study revealed significant differences between drug outlets in Vietnam and Nigeria. In Vietnam, all drug outlets were officially registered and staffed by qualified personnel. In contrast, many outlets in Nigeria were unregistered and often lacked adequately trained staff. Furthermore, the storage conditions in Nigerian outlets were frequently poorly monitored, potentially compromising the stability and potency of the medications. We identified one brand with consistent quality issues, as all preparations showed CIP concentrations that deviated by 36%–54% below the amounts declared on the packaging despite having no dissolution issue in the sample preparation for HPLC. Notably, all seven problematic preparations originated from the same manufacturer in Nigeria and were purchased from different vendors with three different lot numbers, indicating that the quality issues were likely due to manufacturing or quality control deficiencies rather than improper storage.

Substandard and falsified antibiotics are recognized as significant, yet often overlooked, drivers of AMR, particularly in LMICs.^2,8^ In these regions, unregistered and officially sanctioned drug outlets may coexist, and standards for storage and trained staff are not consistently upheld.^9^ Substandard or counterfeit anti-infective drugs can act as catalysts for AMR by exposing pathogens to sub-therapeutic doses during treatment regimens. In many cases, the active pharmaceutical ingredient levels in these drugs are either critically low or entirely absent, rendering the treatment ineffective.^10^ Medications that fail to meet established quality standards—often referred to as substandard medicines—may arise from inadequate manufacturing, shipping or storage conditions, as well as the sale of expired drugs. In contrast, falsified medicines are deliberately misrepresented regarding their identity, composition or origin, typically due to fraudulent activities. In 2017, the WHO reported that approximately 10% of drugs worldwide are falsified, with antibiotics comprising 50% of these cases. Notably, 78% of the reported falsified antibiotics originated from developing countries.^11^

Although regulatory frameworks for quality control exist—such as the National Agency for Food and Drug Administration and Control (NAFDAC) in Nigeria and the Drug Administration of Vietnam (DAV) under the Ministry of Health (MOH)—enforcement remains a major challenge. Both countries have a National Action Plan on AMR, but enforcement appears to be more stringent in Vietnam than in Nigeria. Several challenges hinder effective regulation, including insufficient resources, limited funding and government inefficiencies.^12^

Although both are developing countries, there are notable differences between Vietnam and Nigeria. For example, pharmacies in Vietnam are better regulated, with all randomly sampled outlets registered and staffed by qualified personnel. In contrast, almost half of the drug outlets surveyed in Nigeria were unregistered, highlighting gaps in regulatory oversight. Clearly, policymakers have a pivotal role to play in ensuring stricter enforcement and improving pharmaceutical governance.

A coordinated effort between the national governments of Vietnam and Nigeria and relevant stakeholders is essential to address the problem of substandard and counterfeit medicines effectively. Strengthening the regulatory framework should be a top priority, by enforcing stricter requirements for drug registration and licensing and ensuring standardised training for pharmacy staff. Implementing rigorous quality control measures, such as regular drug testing, thorough monitoring of manufacturers, qualification of suppliers and improved storage practices, is essential to maintain the efficacy of medicines.^12,13^ Raising public awareness through targeted education campaigns can help consumers identify substandard medicines and recognise the risks associated with purchasing medicines from informal markets. More importantly, patients will have access to safe and effective medicines, ensuring the long-term efficacy of essential antibiotics such as CIP and maintaining global health security.

Our study demonstrates that while antibiotics can be purchased over-the-counter without a prescription, most preparations contained the declared antibiotic content and exhibited only minor deviation from the declared content. However, one manufacturer's products showed major deviations from the declared concentrations, underscoring the need for rigorous quality control measures. Despite some limitations—such as the small number of vendors and the limited geographic scope of our study—the findings suggest that substandard antibiotics are not common or representative of the norm in resource-limited settings. Nevertheless, our findings highlight the importance of implementing effective quality control and regulatory measures for antimicrobial drugs, as well as increasing public awareness, to prevent the distribution of substandard antibiotics in order to mitigate AMR in LMICs.

## Supplementary Material

dlaf042_Supplementary_Data

## Supplementary data

Figures S1 and S2 and Table S1 are available as Supplementary data at *JAC-AMR* Online.
