# Supplementary material for: Investigation of CIP quality in over-the-counter drug stores of resource-limited countries: a comparative study in Vietnam and Nigeria
Source: JAC Antimicrob Resist. 2025 Mar 19;7(2):dlaf042. doi: 10.1093/jacamr/dlaf042 (PMC11920618; doi:10.1093/jacamr/dlaf042)
Supplement: dlaf042_Supplementary_Data [file dlaf042_supplementary_data.docx]

**Supplementary Appendix**

**Title:**

**Investigation of ciprofloxacin quality in over-the-counter drug stores resource-limited countries: a comparative study in Vietnam and Nigeria**

**Table of content:**

[**Supplementary Methods** 2](#_Toc191050834)

[**Supplementary Figure S1** 4](#_Toc191050835)

[**Supplementary Figure S2** 5](#_Toc191050836)

[**Supplementary Table S1. Comparison of ciprofloxacin content and purity** 6](#_Toc191050837)

# **Supplementary Methods**

*Ciprofloxacin susceptibility testing*

Standardized disk diffusion was traditionally carried out according to the Kirby-Bauer method based on EUCAST guidelines. Several isolated fresh colonies were picked from nutrient agar and suspended in 0.9% NaCl. The inoculum was adjusted to a turbidity corresponding to a 0.5 McFarland standard visually. Within 15 minutes, a sterile cotton swab was used to streak this inoculum onto Mueller-Hinton agar in three different directions to cover the whole surface of the agar. The appropriate antimicrobial-impregnated disks were placed on the surface of the agar. After incubation at 37°C for 18 hours, the diameters of the inhibition zones were recorded. As a reference, a commercially available disk diffusion test for ciprofloxacin (5µg) was used (Mast Diagnostics, Germany). To test the bacterial activity of the purchased ciprofloxacin preparations, blank disks were impregnated with 5 µg ciprofloxacin and air dried before placing them onto the prepared Mueller-Hinton Agar streaked with 0.5 McFarland *Escherichia coli* ATCC®25922. Quality control for ciprofloxacin disk diffusion (commercial) was performed using *E. coli* ATCC®25922.

*Information on study sites*

This cross-sectional comparative study assessed the quality of ciprofloxacin procured from both authorized and unauthorized pharmacies in Vietnam and Nigeria. In Vietnam, the study was conducted in Thua Thien Hue province, which comprises Hue city, 2 towns (Huong Thuy and Huong Tra), and 6 districts (Phong Dien, Quang Dien, Phu Vang, Phu Loc, A Luoi, and Nam Dong). Hue city is the most densely populated area in the province, with over 40% of the total population of approximately 1.13 million people. In June 2023, samples of oral ciprofloxacin were collected in collaboration with the Drug, Cosmetic, and Food Quality Control Center of Thua Thien Hue province. These samples were randomly obtained from 13 vendors across the province, including 5 vendors in Hue city and 1 vendor in each of the 2 other towns and 6 districts.

In Nigeria, the study took place in Ogbomoso, Oyo State, covering five local government areas (LGAs) (Ogbomoso North, Ogbomoso South, Orire, Ogo-Oluwa, and Surulere). Ogbomoso North is the most populous area in the study region, accounting for over 30% of the local population, approximately 935,000 people. Similar to Vietnam, ciprofloxacin samples were collected randomly from five vendors in Ogbomoso North and 2 vendors in each of the 4 other LGAs in October 2023, with the support of researchers at the Center for Emerging and Reemerging Infectious Diseases, LAUTECH. Figure 1a (in the main text) provides an overview of the characteristics of the study area and the distribution of drug outlets.

The study included drug outlets with all types of businesses, selling oral ciprofloxacin. Pharmacy selections were considered based on the availability of different pharmaceutical brands, dosage forms, and strengths of ciprofloxacin. All available ciprofloxacin samples at each selected pharmacy were then collected for analysis.

*High-Performance Liquid Chromatography*

A 0.025 M phosphoric acid solution was prepared and adjusted to a pH of 2.0 ± 0.1 using triethylamine (TEA). A mixture of acetonitrile and this phosphoric acid solution in a ratio of 13:87 was prepared as Solution B.

For High-Performance Liquid Chromatography (HPLC) analysis. A single 500 mg tablet of ciprofloxacin was placed in a 100 ml volumetric flask. Subsequently, 90 ml of Solution B was added, and the tablet was sonicated for 20 minutes. After sonication, the volume was adjusted to 100 ml with Solution B. The resulting solution was then filtered through a 0.45 µm membrane filter, discarding the first 2 ml, and a final 1 ml aliquot was collected, yielding a concentration of 0.2 mg/ml ciprofloxacin from the filtrate prepared with Solution B. A commercially available ciprofloxacin standard (Sigma Aldrich, Germany) was used for calibration. A stock solution of 1 mg/ml was created by dissolving 1 mg of the ciprofloxacin standard in 1 ml of Solution B. Subsequent dilutions were prepared to obtain concentrations of 0.05 mg/ml, 0.1 mg/ml, 0.2 mg/ml, 0.3 mg/ml, 0.4 mg/ml, and 0.5 mg/ml, all using Solution B.

Analytical reverse-phase HPLC was performed using an Agilent 1260/1290 system, which included a quaternary pump (G1311A), an autosampler (G1329B), a thermostated column compartment (G1316A), a fluorescence detector (G1321A), a diode array detector (G1315C; DAD range λ = 190-400 nm, UV λ = 278 nm), and an analytical fraction collector (G1364C). The separation was carried out on a LiChroCART C18ec column (length: 250 mm, internal diameter: 4 mm, pore size: 100 Å, particle size: 5 μm, Merck). The mobile phases consisted of H3PO4 (HPLC grade, Honeywell Fluka) at 0.025 M, adjusted to pH 3.0 ± 0.1 with TEA, and acetonitrile (B; gradient grade, LiChrosolv, Merck). An isocratic system was employed for the first 30 minutes at 13% B, with a flow rate of 1.5 mL/min. The entire system was managed using Agilent Chemstation (Rev. C.01.05).

**Supplementary Figure S1.** **A** Sample preparation of V11 (QLT037). Ciprofloxacin powder was not dissolvable. **B** 3D plot of QLT037_1. 3D plot shows 2 peaks; peak 1: 190 nm, RT: 1,23 min; peak 2: 278 nm, RT:10,342 min. **C** Chromatogram of QLT037_1. Chromatogram shows a peak at 278 nm. RT: 10,342 min. **D** Chromatogram of QLT018_1. Chromatogram shows a peak at 278 nm, RT. RT: 10,388 min.


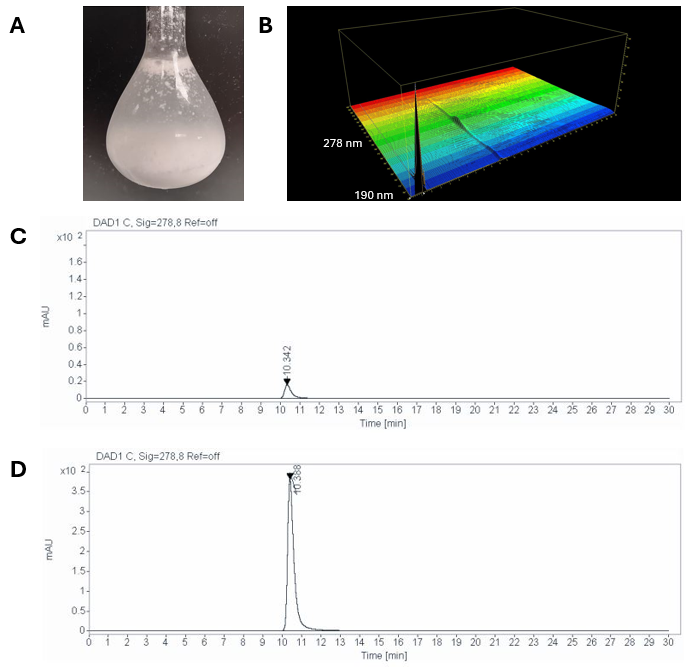


**Supplementary Figure S2.** **A** Sample preparation of N4 (QLT004). The tablet QLT004_2 has a brownish color in contrast to the other tablets. **B** 3D plot of QLT004_1. 3D plot shows a peak at 278 nm, RT: 10,291 min. **C** Chromatogram of QLT004_1. Chromatogram displays a peak at 278 nm, RT 10,291 min. **D** 3D plot of QLT004_2. 3D plot shows two peaks. Peak 1: 246 nm, RT: 1,424 min; Peak 2: 278 nm, RT: 10,195 min. **E** Chromatogram of QLT004_2. Chromatogram shows two peaks. Peak 1: RT 1,424 and Peak 2: RT 10,393; 278 nm.


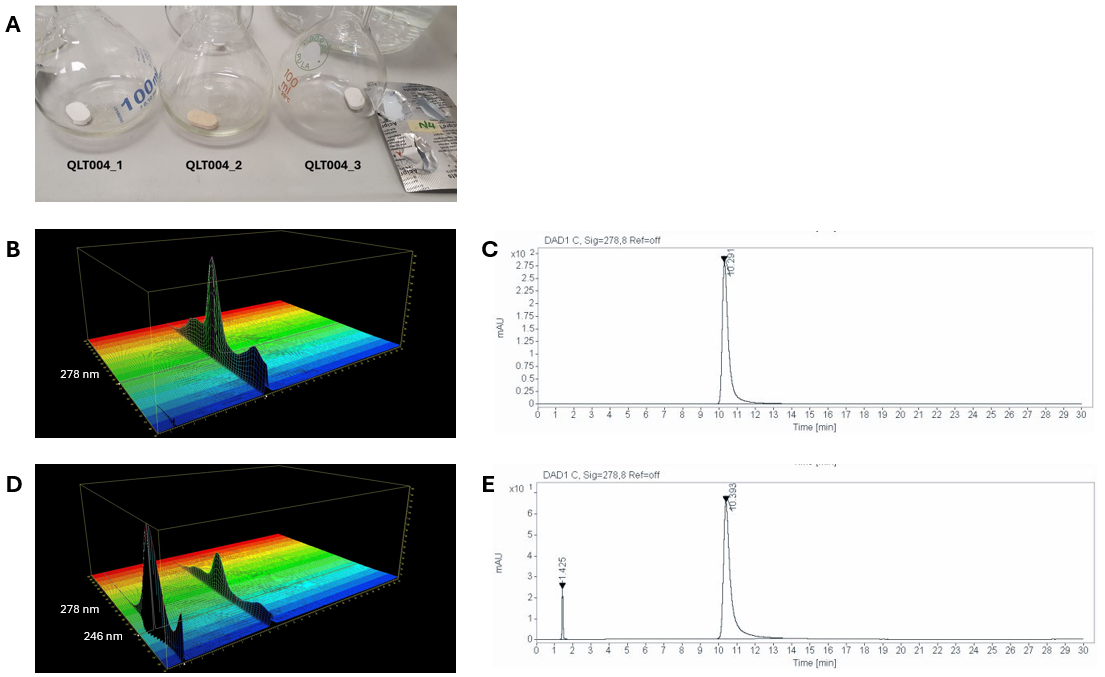


**Figure 1 QLT004**

# **Supplementary Table S1. Comparison of ciprofloxacin content and purity**

| **Sample code** | **Brand name** | **company/manufactured by** | **origin** | **Lot number** | **Substance (API)** | **concentration according to packagin** | **concentration measured [mg]** | **Percent of declared content [%]** | **Deviation from declared content [%]** | **purity [%]** |
| --- | --- | --- | --- | --- | --- | --- | --- | --- | --- | --- |
| N1 | Brand 1 | Manufacturer A | Nigeria | ACI23128 | Ciprofloxacin Hydrochloride | 500 | 320.833 | 64% | 36% | 100 |
| N2 | Brand 1 | Manufacturer A | Nigeria | ACI23065 | Ciprofloxacin Hydrochloride | 500 | 305 | 61% | 39% | 100 |
| N3 | Brand 1 | Manufacturer A | Nigeria | ACI23128 | Ciprofloxacin Hydrochloride | 500 | 231.699 | 46% | 54% | 100 |
| N4 | Brand 1 | Manufacturer A | Nigeria | ACI23053 | Ciprofloxacin Hydrochloride | 500 | 245 | 49% | 51% | 97.62 |
| N5 | Brand 1 | Manufacturer A | Nigeria | ACI23128 | Ciprofloxacin Hydrochloride | 500 | 319.167 | 64% | 36% | 100 |
| N6 | Brand 1 | Manufacturer A | Nigeria | ACI23128 | Ciprofloxacin Hydrochloride | 500 | 308.922 | 62% | 38% | 100 |
| N7 | Brand 1 | Manufacturer A | Nigeria | ACI23128 | Ciprofloxacin Hydrochloride | 500 | 330.85 | 66% | 34% | 100 |
| N8 | Brand 2 | Manufacturer B | Nigeria | CV41 | Ciprofloxacin Hydrochloride | 500 | 452.617 | 91% | 9% | 100 |
| N9 | Brand 2 | Manufacturer B | Nigeria | CV44 | Ciprofloxacin Hydrochloride | 500 | 459.558 | 92% | 8% | 100 |
| N10 | Brand 2 | Manufacturer B | Nigeria | CV39 | Ciprofloxacin Hydrochloride | 500 | 442.01 | 88% | 12% | 100 |
| N11 | Brand 2 | Manufacturer B | Nigeria | CV37 | Ciprofloxacin Hydrochloride | 500 | 460.524 | 92% | 8% | 100 |
| N12 | Brand 2 | Manufacturer B | Nigeria | CV42 | Ciprofloxacin Hydrochloride | 500 | 447.302 | 89% | 11% | 100 |
| N13 | Brand 3 | Manufacturer C | India | CO12007 | Ciprofloxacin Hydrochloride | 500 | 438.324 | 88% | 12% | 100 |
| N14 | Brand 3 | Manufacturer C | India | CO12003 | Ciprofloxacin Hydrochloride | 500 | 460.82 | 92% | 8% | 100 |
| N15 | Brand 3 | Manufacturer C | India | CO12016 | Ciprofloxacin Hydrochloride | 500 | 435.615 | 87% | 13% | 100 |
| N16 | Brand 3 | Manufacturer C | India | CO13004A | Ciprofloxacin Hydrochloride | 500 | 445.718 | 89% | 11% | 100 |
| N17 | Brand 4 | Manufacturer D | India | AC95521 | Ciprofloxacin Hydrochloride | 500 | 453.34 | 91% | 9% | 100 |
| N18 | Brand 5 | Manufacturer E | China | 220903 | Ciprofloxacin Hydrochloride | 500 | 447.275 | 89% | 11% | 100 |
| N19 | Brand 6 | Manufacturer F | China | 210203 | Ciprofloxacin Hydrochloride | 500 | 441.52 | 88% | 12% | 100 |
| N20 | Brand 7 | Manufacturer G | India | BA-7154 | Ciprofloxacin Hydrochloride | 500 | 410.209 | 82% | 18% | 100 |
| N21 | Brand 8 | Manufacturer H | Nigeria | T2521009 | Ciprofloxacin Hydrochloride | 500 | 443.765 | 89% | 11% | 100 |
| N22 | Brand 9 | Manufacturer I | China | 230436 | Ciprofloxacin Hydrochloride | 500 | 427.016 | 85% | 15% | 100 |
| N23 | Brand 10 | Manufacturer J | India | TE1295 | Ciprofloxacin Hydrochloride | 500 | 441.063 | 88% | 12% | 100 |
| N24 | Brand 11 | Manufacturer H | Nigeria | CA 0017 | Ciprofloxacin Hydrochloride | 500 | 436.706 | 87% | 13% | 100 |
| N25 | Brand 12 | Manufacturer K | Nigeria | A222061 | Ciprofloxacin Hydrochloride | 500 | 471.441 | 94% | 6% | 100 |
| N26 | Brand 13 | Manufacturer K | Nigeria | AC23014 | Ciprofloxacin Hydrochloride | 500 | 457.199 | 91% | 9% | 100 |
| V1 | Brand 14 | Manufacturer L | Vietnam | 00423 | Ciprofloxacin Hydrochloride | 500 | 459.351 | 92% | 8% | 100 |
| V2 | Brand 15 | Manufacturer M | Vietnam | 020522 | Ciprofloxacin Hydrochloride | 500 | 459.589 | 92% | 8% | 100 |
| V3 | Brand 16 | Manufacturer N | India | BNT1022018 | Ciprofloxacin Hydrochloride | 500 | 448.509 | 90% | 10% | 100 |
| V4 | Brand 17 | Manufacturer O | India | FCF2191A | Ciprofloxacin Hydrochloride | 500 | 442.984 | 89% | 11% | 100 |
| V5 | Brand 18 | Manufacturer P | Vietnam | 0050222 | Ciprofloxacin Hydrochloride | 500 | 455.745 | 91% | 9% | 100 |
| V6 | Brand 19 | Manufacturer Q | Cyprus | A6L119 | Ciprofloxacin Hydrochloride | 500 | 458.402 | 92% | 8% | 100 |
| V7 | Brand 20 | Manufacturer R | Vietnam | 00622 | Ciprofloxacin Hydrochloride | 500 | 455.467 | 91% | 9% | 100 |
| V8 | Brand 21 | Manufacturer S | Vietnam | 051222 | Ciprofloxacin Hydrochloride | 500 | 454.707 | 91% | 9% | 100 |
| V9 | Brand 22 | Manufacturer T | Vietnam | 03022023 | Ciprofloxacin Hydrochloride | 500 | 454.463 | 91% | 9% | 100 |
| V10 | Brand 23 | Manufacturer U | Vietnam | 071022 | Ciprofloxacin Hydrochloride | 500 | 472.443 | 94% | 6% | 100 |
| V11 | Brand 24 | Manufacturer V | Vietnam | 011122 | Ciprofloxacin Hydrochloride | 250 | 11.365 | 2% | 95% | 100 |
| V12 | Brand 25 | Manufacturer W | Germany | BXJHJF2 | Ciprofloxacin Hydrochloride | 500 | 432.881 | 87% | 13% | 100 |
| V13 | Brand 26 | Manufacturer X | India | MOSHO161 | Ciprofloxacin Hydrochloride | 500 | 465.119 | 93% | 7% | 100 |
| V14 | Brand 26 | Manufacturer X | India | MOSH0135 | Ciprofloxacin Hydrochloride | 500 | 472.138 | 94% | 6% | 100 |
| V15 | Brand 27 | Manufacturer Y | Vietnam | 041222 | Ciprofloxacin Hydrochloride | 500 | 467.88 | 94% | 6% | 100 |
| V16 | Brand 27 | Manufacturer Y | Vietnam | 030422 | Ciprofloxacin Hydrochloride | 500 | 437.965 | 88% | 12% | 100 |
| V17 | Brand 27 | Manufacturer Y | Vietnam | 041222 | Ciprofloxacin Hydrochloride | 500 | 444.529 | 89% | 11% | 100 |
| V18 | Brand 27 | Manufacturer Y | Vietnam | 041222 | Ciprofloxacin Hydrochloride | 500 | 446.981 | 89% | 11% | 100 |
| V19 | Brand 28 | Manufacturer Z | Vietnam | 050822 | Ciprofloxacin Hydrochloride | 500 | 458.262 | 92% | 8% | 100 |
| V20 | Brand 29 | Manufacturer AA | Vietnam | 23003 | Ciprofloxacin Hydrochloride | 500 | 436.078 | 87% | 13% | 100 |
